# Round the Hospitals

**Published:** 1920-06-12

**Authors:** 


### ROUND THE HOSPITALS.

A meeting which resolved itself into a debate on the rival claims of the College of Nursing, and the Royal British Nurses Association was held on Saturday evening at the London Temperance Hospital. Many nurses of the hospital were present, and a sprinkling of visitors. The objects of the College and the ideals for which it stands were explained by Miss Sheriff Macgregor, R.R.C., its organising secretary, in a written paper which, owing possibly to its necessary brevity, lacked something in lucidity. She was opposed by Miss Isabel Macdonald, secretary of the Royal British Nurses Association, who concerned herself chiefly with the past history of the Association. She

**ROUND THE HOSPITALS—(*continued*).**

argued that the matrons stood in the way of nurses joining the Association, owing to its democratic basis, but if they gave it better support it would sweep all before it. The chair was taken by Mr. H. Paterson, F.R.C.S., hon. secretary of the R.B.N.A., who was so conspicuously fair to both societies that it would have been impossible for an outsider to know which he represented. He did much to keep the peace in the rather ill-natured discussion which followed the speeches. Perhaps the most useful contribution to the evening's proceedings was the little speech made by Miss Donaldson, matron of the London Temperance Hospital, who said that nothing pleased her better than to find her nurses in opposition to her and to hear their opinions. She liked to feel that they had strong views and were ready to express them. She urged all her nurses present to think carefully over all that had been said and then to take action. Perhaps the only time when the meeting was in agreement was when a member of the audience expressed a wish that more matrons were like Miss Donaldson. The College of Nursing was sparsely represented.

The event of next week will be the Annual Meeting of the College of Nursing, which is fixed for Thursday, June 17, and will be held at the Hall of the Royal Society of Medicine, 1 Wimpole Street, W., at 3 P.M. Only members can obtain admission, and they should come in good time, for a record attendance is already foreshadowed. The company will be welcomed when the business is over at 7 Henrietta Street, and may obtain tea tickets (price 1s.) at the hall. We are glad to think there is a prospect that next year the meeting may be held in the members' own commanding premises. The time has fully arrived when the College should be fitly housed, and the requirements of the London Centre greatly emphasise the necessity of the much-desired headquarters which shall be worthy of the College.

The conferences which succeed the annual meeting of the College of Nursing have become an important feature in this unique nursing festival. A discussion on "How to attract the most suitable women to the nursing profession" is to take place on June 17 at 8 P.M. in the hall at 1 Wimpole Street. The Chairman is Dr. J. Kay Jamieson, and the speakers are Miss J. Lane-Claypon, M.D., Dean, Household and Domestic Science Department, King's College for Women; Miss Musson, R.R.C., General Hospital, Birmingham; and Miss Edmondson, R.R.C., while Miss Montgomery, R.R.C., will be in charge of the session, a circumstance which ensures its smooth and successful tenor. On Friday morning, June 18, at 11, a conference will take place on "District Nursing," in which Miss E. Hancox, Miss G. R. Skinner, Miss Tait-McKay, Miss Hughes, Miss Peterkin, and Miss Grace Vaughan will take part, with Sir William Cameron Gull, O.B.E., in the chair. A great number of nurses are coming up for the meeting and conferences from various parts of the kingdom and are obliged to get back in the afternoon of the 18th, so that it is necessary to have the second conference in the morning. It will, at any rate, be a very convenient hour for night nurses before they turn in, and we trust more leisured members of the profession will not neglect this opportunity of hear- a weighty subject handled from the nurses' own point of view as well as from that of the nation.

There is very good news in the Treasurer's report of the Royal Hospital of St. Barthlomew for last year, which has just come to hand, of the projected Nurses' Home. The plans have been approved and adopted by the Court of Governors, and comprise four blocks facing respectively south, south-east, east, and north-west. Of these one block, the south, is to erected forthwith; it demands no interference with the present accommodation, and only one staircase of the old Christ Hospital building has to be removed. This block will accommodate 116 persons and can be arranged to provide temporary bedrooms for 194. Its cost is estimated at £80,000, and the amount received or promised to date is only £7,000 short of this sum. For a donation of £350 the donor has the privilege of naming a room in the Home. The architect's general plan of construction is of considerable interest and may be studied as an example of the highest point yet reached in the planning of such establishments. Bath-rooms are to be in proportion one to six to one to seven bed-rooms; water-closets in proportion one to five to one to six. Rooms are provided on each floor for the following purposes: shampoo, linen, box, boot-cleaning, cloak-drying, tea-making, besides lifts for passengers and service, trolley stores and cleaners rooms. The bedrooms are to be normally twelve by eight feet clear and will have fixed furniture. Each of the four blocks will be furnished with a flat roof and afford space for a roof-garden.

The matron of St. Bartholomew's received 1,228 applications for regulations from candidates last year, as compared with 851 in 1918, a sign that matters are righting themselves, and added twelve additional probationers to the staff, for whom temporary sleeping quarters have been provided by fitting up cubicles in the old surgery. Sixty nurses completed three years' training and gained their certificates. The total number of St. Bartholomew's nurses who served in the war is estimated at 350. Of these 111 received honours and 158 were mentioned in despatches. Among the honours gained were seven Bars, thirty-nine 1st Class, and fifty-three 2nd Class Royal Red Cross. The number of nurses on the private staff who work on the co-operative system, receiving their own fees less seven and a-half per cent. for working expenses, is at present somewhat below the normal average of 75.

The ninth edition of the Guy's Hospital *Nursing Guide* just issued is an admirable production which reflects high credit on all concerned in its preparation. Its main purpose is to provide a hand-book

**ROUND THE HOSPITALS—*(continued)*.**

of the Guy's Nurses' League and a Register of the nurses trained at the institution, but it has earned for itself a place in the nursing world irrespective of this particular training school. The articles on "The Nursing Profession of To-day," on private and district nursing are well thought out and modern, while the particulars furnished of the gymnastic and massage, medical electricity and radiography departments of the hospital are of great value. We are glad to see a *résumé* of the National Health Insurance Acts among the information provided in the body of the book. There are many useful nursing instructions, and formulæ with a capital section devoted to invalid cookery. The Register itself contains a remarkably full account of the career of each nurse and is likely to be of continued service to nurses as a supplement to State Registration. The list of honours gained by Guy's nurses in the war has been brought up to date and is particularly interesting. It numbers one bar, twenty-nine first class and eighty-six second class of the Royal Red Cross, besides many other decorations and a long list of "mentions."

---

The Guy's Hospital Nurses' League, which lately held its annual meeting, is undoubtedly, quite irrespective of the date when it was founded, a pioneer. Its wealth of activities, its library, swimming club, musical, dramatic, photographic, and debating societies, its needlework exhibitions and competitions, its gardens, cycling club, and cottage for rest and convalescence, its tennis, and other forms of organised recreations have made it an example for leagues in every part of the kingdom. The local centres of the College of Nursing could not do better when in search of ideas than take lessons from Guy's nurses, but the secret of their live, purposeful off-duty existence does not lie on the surface. It has been a gradual growth. It owes much to the initiative of successive, far-seeing matrons; it has been fostered with pride and generosity by the lay managers; but best of all it triumphantly demonstrates the fact that success in this direction lies in the spontaneous efforts of nurses themselves, directing their own affairs. There has never lacked talent often of a brilliant order in whatever line it was required. The result is an object-lesson in the variety of latent powers possessed by women with a vocation.

---

London nurses should not fail to look in at the College of Ambulance 56 Queen Anne Street, W. 1, to see the wonderful allegorical painting entitled "Nurse Cavell or the Scrap of Paper," now on view there. The artist is Mr. Horace Van Ruith, and special interest attaches to this picture from the fact that he painted it when partially blind.

---

The Duchess of Bedford's surgical hospital at Woburn, where treatment was extended for many months after the war to wounded soldiers, has now closed down. On the outbreak of war the building was a riding-school, and it was promptly equipped by the Duchess, who devoted a great part of her time to work in the wards. She took spells of duty as one of the regular staff, starting work often at 6 A.M., and on occasions put in as many as sixteen hours a day. Her interest in the patients led her to make arrangements for their friends to come up and stay near at hand, and she took pains in countless ways to lessen the after difficulties of all who came under her care. It was a fine example, and one which has borne fruit.

---

We cannot forbear pointing out once again the absurdity of the messing arrangements in Poor-Law Infirmaries. At a recent meeting of the Camberwell Guardians Mrs. Morris presented a report from the Infirmaries' Committee recommending that "with a view to a little extra being provided the nurses for tea on Sundays the matron be allowed to requisition after the rate of an additional twopence per head per week in respect of same." The Board demurred to this on the ground that it was costing 16s. per head per week at contract prices to feed the nurses. It must be evident to all sensible persons that were the messing arrangements under the control of an experienced woman with full liberty to order what was necessary within the limits of a weekly average of 16s. per head, ample variety could be provided not only for Sunday tea, but for Monday and all the days of the week. But official jealousy dictates that all must be worked out beforehand, and the number of pounds of every commodity to be bought dictated to the steward, quite irrespective of the individual tastes of the people who have to eat the food. If a proper working system of housekeeping as in voluntary institutions were adopted a weekly average of 15s. or 15s. 6d. per head would certainly suffice. The staff could be very liberally catered for and the rate-payers would stand to gain.

---

The nurses at the Sydney Hospital are very unhappy at the decision of the Board of Directors to move them out of their quarters at the hospital to a house situated at some little distance in a quarter known as Woolloomooloo. The nurses have petitioned the Board against the change and express their strong objection to the sordid outlook and unpleasant surroundings of the place selected for them. As many as 100 out of a nursing staff of 130 are likely to resign if the project is carried through. In the same city a beautiful nurses' home has recently been opened at the Woman's Hospital. It is built in a Spanish-American style, with a flat roof garden, broken by shelter kiosks, commanding a magnificent view. There are forty-seven bedrooms, most of which open direct on to balconies, and all have been charmingly furnished in oak by private donors. Wise provision has been made for further expansion. It is a great pity that the authorities at the Sydney Hospital have not seriously tackled the question of building a new Nurses' Home after this good example instead of renting premises distasteful to their staff.

**ROUND THE HOSPITALS**—(*continued*).

At the annual prize-giving of the Royal Devon and Exeter Hospital Mrs. Stirling, wife of the President, Brigadier General Stirling, C.B., C.M.G., presented the gold medal to Nurse Harrison for excellence in surgical, nursing, medical, special, and ward work, and the silver medal to Nurse Brocklehurst, who was only a few points behind. Twelve other nurses obtained passes. The chairman made an interesting point in stating that every single member of the nursing staff had been trained in the hospital, the matron, Miss Small, having once been a probationer. It is a very uncommon condition of affairs, and the more remarkable because during the war the staff was considerably depleted. Sympathetic reference was made to the nursing sister who was killed on a torpedoed hospital ship. The high level of technical skill possessed by the nurses was commented on with appreciation by General Stirling, who spoke warmly of the noble way in which they had risen to their opportunities during the war. A pleasant reception with tea in the hospital grounds followed.

Certificate Day at Crumpsall Infirmary is always a pleasant function, and this year it was no exception to the rule. The distribution of the recent final nurses' certificates took place on Thursday, June 3, in the recreation room. In addition to as many nurses as could be spared from the wards, the parents and friends of those receiving their certificates were present. The three nurses who headed the list received prizes in the shape of books, given respectively by the chairman, the matron of Booth Hall Infirmary, and the chaplain of the infirmary. The Hon. R. H. Cawley, chairman of the Infirmary Committee, congratulated the sixteen nurses who were receiving certificates, and particularly three who had sat for the second time and passed. Mr. Cawley emphasised the desire of the guardians to keep up the traditions of the infirmary as a training-school. The latest instance of this aim is the appointment of a sister-tutor, who, it is hoped, will take up her duties in August. At the invitation of the matron tea was served after this distribution, and the day was further celebrated by dancing in the evening, the recreation room being charmingly decorated with plants and flowers.

The enormous rise of prices by reason of the war has seldom been more forcibly presented than in the treasurer's report of St. Bartholomew's Hospital. We quote some of the figures and have given (1) the contract price in 1913-14, and (2) the average price in 1919: Beef per stone eight pounds (1) 3s. 4d.; (2) 11s. 10d. Mutton-legs (1) 3s. 4d.; (2) 11s. 5d. Suet (1) 4s.; (2) 11s. 0½d. Margarine per cwt. (1) £2 9s.; (2) £5 9s. 6½d. Cheese per cwt. (1) £3 18s.; (2) £7 19s 10d. Bacon per cwt. (1) £4 2s.; (2) £10 17s. 2d. Bread per cwt. (1) 10s. 6d.; (2) 18s. 4d. Flour per 280 lb. (1) £1 8s.; (2) £2 7s. 6d. Milk per gallon (1) 9¾d.; (2) 2s. 5¼d. Is it any wonder that institutions find themselves reduced to penury?

Influenza was responsible for an increase of £1,000 in the amount paid out by the Nurses' Insurance Society in sickness and disablement benefits last year. The Vice-Chairman, Mr. Frederick Schooling, speaking at the annual meeting, urged upon nurses in their own interests to be more businesslike in reading the notices sent them from time to time, in extinguishing their arrears when applied to, and in applying for benefit within three days from the beginning of an illness. Sickness benefit will shortly rise from 7s. 6d. a week to 12s., and disablement allowance from 5s. to 7s. 6d., in accordance with the new Act, which comes into force on July 5. Is it asking too much of the training-schools to help new probationers to understand their obligations and privileges in this respect?

The scarcity of nurses in the four leading hospitals at Sheffield is creating much dislocation and pressing very hardly on the depleted staffs. There is a strong movement in favour of adopting the College of Nursing scale of pay in them all. The nurses' and sisters' rates of pay are compared in the local press with that offered to nursery maids, housemaids, and cooks, and the conclusion reached is that the proposed revision of pay should come into operation at the earliest possible date. The main thing, according to the *Sheffield Telegraph*, is to make Sheffield "financially attractive to nurses." We like this expression.

The York Maternity Hospital is evidently growing in favour with its clientèle. The number of cases admitted rose from 143 in 1916 to 239 in 1919, in which year £594 was contributed by patients in varying amounts, the average weekly cost of treatment and maintenance being £3 3s. With only fifteen beds the hospital is now quite inadequate to cope with the demands made on it, and is compelled to refuse pre-natal, septic, or venereal cases. A large extension is forecasted, divided into separate wings with at least fifty beds altogether. It is probable that the East and North Riding County Councils would help substantially in the upkeep by payments for cases admitted from outlying villages. A large teaching and training maternity centre in York would be of the greatest service to the entire county by facilitating the output of nurse-midwives.

The Suffolk County Nursing Association, after twenty-four years' work, has followed the lead given in Sussex and has split up into two divisions, East and West. This decision was arrived at on May 20, when, at the annual meeting, executive committees were appointed respectively for East and West Suffolk. Unfortunately nine district societies have had to suspend work owing to the lack of nurses. Many new ones, however, are starting, and there are fifty-five altogether in working order. Sixteen scholarships were granted, but only eight nurses continued their studies to the end.

